# Depressive Symptoms among Patients with Heart Failure in Korea: An Integrative Review

**DOI:** 10.3390/healthcare4030052

**Published:** 2016-08-04

**Authors:** Boyoung Hwang, Heeseung Choi

**Affiliations:** College of Nursing & Research Institute of Nursing Science, Seoul National University, 103 Daehak-ro, Jongno-gu, Seoul 03080, Korea; hchoi20@snu.ac.kr

**Keywords:** depression, heart failure, Korea, integrative review

## Abstract

This integrative review was conducted to examine studies reporting depressive symptoms among patients with heart failure (HF) in Korea. An extensive search with both English and Korean search terms was conducted using six electronic databases. Publications were screened by both authors independently, and 10 articles meeting the inclusion criteria were reviewed. All 10 studies were data-based, quantitative, and descriptive in nature. In all studies, depressive symptoms were measured at only one point in time. The prevalence of depression reported in these studies ranged from 24% to 68%. Heterogeneity in the study samples and measures of depression was noted. Depressive symptoms have received limited attention in research with HF patients in Korea. Additional studies, especially longitudinal studies and intervention studies, are needed to assess depressive symptoms and to test the effects of pharmacological and non-pharmacological interventions on depression among patients with HF in Korea. Clinicians need to screen patients with HF for depressive symptoms using validated measures and provide proper treatment for those who are depressed.

## 1. Introduction

Approximately, 5.7 million individuals in the U.S. [1] and more than 15 million people in Europe [2] currently suffer from heart failure (HF). The numbers are expected to grow with the improvement in the treatment of cardiovascular disease and the aging of the population [1,3]. Unfortunately, there has been no report on the actual prevalence of HF in Korea, but it has been estimated that approximately 0.62% of the Korean population might be living with HF based on data from the National Health Insurance Corporation in 2010 [4]. The actual prevalence may be much higher than this estimate based on sample data [4], and the incidence and prevalence of HF in Korea are expected to rise with the rapid aging of society.

HF is a chronic debilitating disease and patients experience a variety of symptoms that limit their activities of daily living, such as dyspnea, fatigue, and edema [5]. The chronic nature of the disease and its negative influence on daily activities are thought to contribute to the high prevalence of depression among patients with HF, which has been reported to range from 11% to 42% in studies conducted in the U.S., Canada, and Europe [6]. In addition, pathophysiological mechanisms, such as neurohormonal activation, inflammation, cardiac dysrhythmias, and altered platelet function, which are known to contribute to the development and progression of both HF and depression, may also explain the high prevalence of depressive symptoms among patients with HF [7,8].

Compared to patients with HF who are not depressed, those who are depressed are less likely to carry out self-care activities that are important in managing HF symptoms and preventing the exacerbation of symptoms [9]. Depression has also been found to be a strong, independent predictor for negative outcomes in patients with HF, such as cardiac events, readmission, and mortality [6,10]. Despite the well-known detrimental effects of depressive symptoms on outcomes of patients with HF, little attention has been given to this issue in Korea. Moreover, differences have been reported across cultures in the prevalence of depression (e.g., lower prevalence rates were found in Asian cultures than in Western cultures) and in the presentation of symptoms (e.g., somatic depressive symptoms were more frequently reported in non-Western cultures than in Western cultures) [11]. Therefore, in order to guide clinical practice and future research in Korea, a review of the evidence on depressive symptoms among patients with HF with a focus on the Korean population is needed. In addition, reviewing articles written in both English and Korean will provide a comprehensive understanding of the current literature. To address these gaps in the research, we conducted an integrative review of the literature to examine studies reporting depressive symptoms among patients with HF in Korea. The specific aims were to: (1) synthesize the evidence on depressive symptoms among patients with HF in Korea; and (2) identify gaps in the literature to provide guidance for future studies aiming to reduce depressive symptoms and improve outcomes of patients with HF in Korea.

## 2. Methods 

Following the integrative review methodology outlined by Whittemore and Knafl [12], a search was conducted to identify relevant articles published by 12 February 2016. The following electronic databases were used: PubMed, EMBASE, Cumulative Index to Nursing and Allied Health Literature (CINAHL), Korean Medical Database (KMbase), Research Information Sharing Service (RISS), and Korean Studies Information Service System (KISS). Search terms included “heart failure” and “Korea”, in combination with each of the following search terms: “depression”, “depressive symptoms”, or “psychological distress”. For the following databases, terms were entered in both English and Korean: KMbase, RISS, and KISS. The two authors (B.H. and H.C.) independently screened publications using the following inclusion criteria: (1) reporting depressive symptoms of patients with HF in Korea; (2) peer-reviewed; and (3) published in either English or Korean. In addition, all studies were required to report depressive symptoms in a sample composed exclusively of patients diagnosed with HF or report depressive symptoms specifically for a subgroup of patients with HF. The two authors independently extracted data on the following study characteristics from each study: study design, sample characteristics (e.g., age, gender, severity of HF), recruitment setting characteristics (i.e., in-patient vs. out-patient settings), assessment methods of depression, and main findings (e.g., prevalence of depression). To ensure data accuracy, data were rechecked against the original articles. The authors discussed any discrepancies until an agreement was reached.

## 3. Results

### 3.1. Study Characteristics 

**Figure 1:**
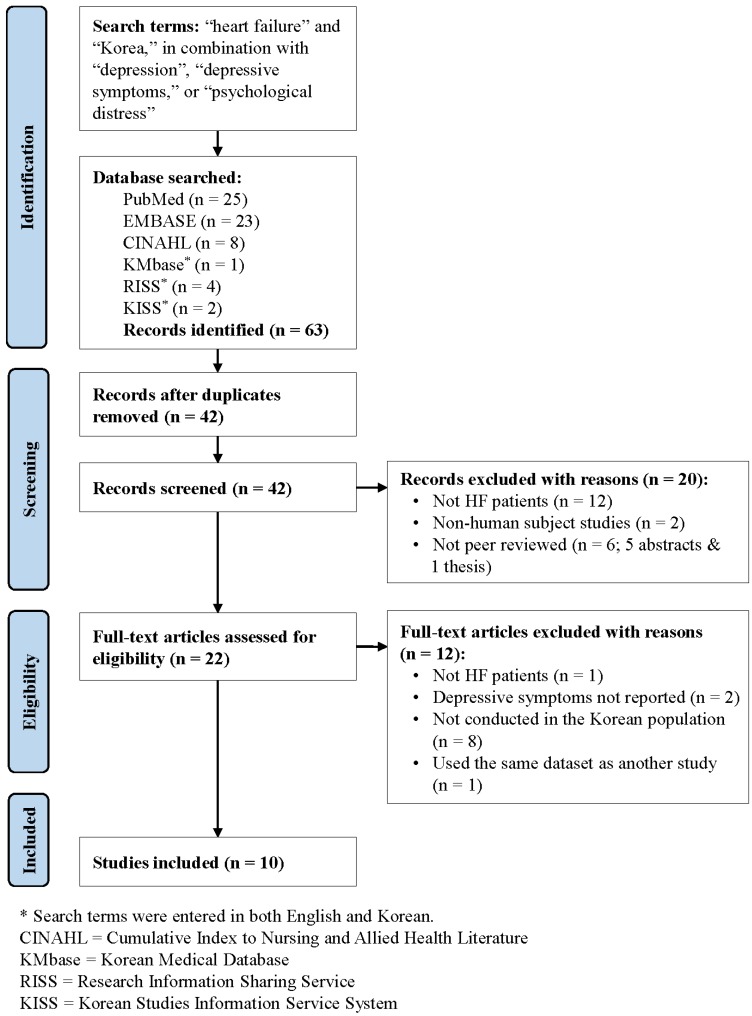
Study identification process.

As indicated in Figure 1, the initial search yielded 63 publications. After deleting duplicate publications (*n* = 21), the remaining 42 publications were screened using the aforementioned criteria. An additional 20 publications were excluded at this stage, and 22 full-text articles were assessed for eligibility. This process yielded a final sample of 10 studies. 

Table 1 summarizes the key characteristics of the remaining 10 studies that met the eligibility criteria for the review. The publication years ranged between 2005 and 2015, and six of the 10 studies were published within the last five years. All studies were data-based (i.e., no review articles), quantitative (i.e., no qualitative studies), and descriptive in nature (i.e., no intervention studies). Six studies were cross-sectional, and four were prospective. However, in the four prospective studies [13,14,15,16], depressive symptoms were measured only once, and therefore no data were reported on changes in depressive symptoms over time among patients with HF in Korea. The mean age of patients with HF enrolled in these studies ranged from 61 [14,17] to 68 years [18]. The study samples varied in terms of gender, severity of HF, and recruitment setting. The percentage of female patients ranged from 20% [19] to 63% [18]. The percentage of patients with a New York Heart Association (NYHA) class of III or IV, indicating moderate to severe HF symptoms, also varied greatly, ranging from 10% [17,19] to 66% [20]. Patients were recruited from in-patient settings in three studies [14,20,21], whereas the remaining seven studies included patients with HF who were being treated in outpatient settings. 

### 3.2. Depressive Symptoms 

In all the studies reviewed, depressive symptoms were measured using self-report questionnaires and no other methods, such as diagnostic interviews, were used. Four measures of depressive symptoms were identified: the Beck Depression Inventory (BDI) [13,14,20,21], Center for Epidemiologic Studies Depression Scale (CES-D) [19,22], Geriatric Depression Scale-Short Form (GDS-SF) [18], and Patient Health Questionnaire (PHQ-9) [15,16,17]. In eight studies [13,14,15,16,17,18,19,21], the prevalence of depression was reported, which ranged from 24% [15] to 68% [18]. However, the cut-off points used to determine the prevalence of depression were not applied consistently across studies. Among the studies that measured depressive symptoms with the BDI, patients were considered to be clinically depressed when their score was 16 or greater in one study [13], but a score of 17 or greater was considered as clinical depression in the other two studies [14,21]. Similarly, a cut-off score of 10 was used in the two studies with the PHQ-9 [15,16], while a cut-off score of 5 was used in another study with the same measure [17]. Discrepancies were also found between the two studies with the CES-D [19,22] in the number of items in the measure and the scoring methods used. H. Lee et al. [19] reported using the 20-item CES-D and indicated that scores on each item could range from 0 to 3, and therefore the total scores could range from 0 to 60. However, S. Lee and colleagues [22] reported using the 16-item CES-D and indicated that each item score could range from 1 to 4, resulting in total scores ranging from 16 to 64. 

In a few studies, the percentage of patients who were receiving antidepressant treatment was reported. One study with 231 hospitalized patients with HF, in which 45% of the study sample were found to be clinically depressed, reported that 26% of the study sample were taking antidepressants [21]. In another two studies with patients with HF who received care in outpatient settings, 48% and 31% of the study samples were clinically depressed, respectively, while antidepressant use was reported in 14% and 25% of the study samples, respectively [13,16]. 

## 4. Discussion

To our knowledge, this is the first integrative review of depressive symptoms among patients with HF in Korea. The growing prevalence of HF worldwide and the well-known negative impact of depressive symptoms on patient outcomes highlight the need to assess depressive symptoms in patients with HF and to provide proper care for those who are depressed. Nevertheless, there are only a limited number of studies that have reported depressive symptoms of patients with HF in Korea. Through a comprehensive literature search, we identified 10 descriptive studies, but were unable to find any qualitative or intervention studies, all of which clearly indicates the limited attention given to this problem in the country. 

The depressive symptoms of patients with HF were measured only once in all 10 studies, and therefore, there is no data currently available on how the depressive symptoms of Korean patients with HF change over time. In a longitudinal study with 457 patients with HF conducted in the U.S., 28% of the patients were found to be clinically depressed at baseline [10]. When depressive symptoms were measured again at 12 months, 41% of the depressed patients had remitted, and 12% of those who were not depressed at baseline had developed depressive symptoms. Similarly, following 611 patients with HF in Europe over 18 months, Johansson et al. [23] reported that 38% were depressed at baseline and that 61% of these patients experienced remission within 18 months. In addition, 19% of those who were not depressed at baseline had developed clinical depression 18 months later. As these data show, depressive symptoms fluctuate considerably over time, and therefore, measuring depressive symptoms at one point in time does not provide the full picture. Future studies need to include longitudinal data on depressive symptoms among patients with HF in Korea. 

The prevalence of clinical depression among patients with HF was reported in eight studies [13,14,15,16,17,18,19,21]. The prevalence rate ranged from 24% to 68%, which was similar to the prevalence reported in studies conducted in Western countries [6]. The wide range may be attributed to heterogeneity in the study samples and measures of depressive symptoms. First, there was wide variability in the demographic (i.e., gender) and clinical characteristics (i.e., NYHA class) of the samples and recruitment settings (i.e., in-patient vs. out-patient settings). Although HF affects men and women equally [1], less than 30% of the study sample was female in some of the studies included in this review [15,17,19]. In three studies [17,19,22], less than 15% of the study sample had a NYHA class of III or IV. Because depression is more common in women than in men and patients with more severe HF symptoms tend to have more severe depressive symptoms [6], the wide variability in the prevalence rates may be explained by these different characteristics of the study samples. Out of the 10 studies reviewed, three studies [14,20,21] measured depressive symptoms among hospitalized patients with HF. In a meta-analysis of 36 studies on depression and HF, which were conducted in the U.S., Canada, and Europe, Rutledge et al. [6] found no significant differences in the prevalence of depression between inpatient and outpatient samples. Due to the small number of studies included in this review, we were unable to conduct a quantitative analysis. However, the prevalence rates of depression reported in the studies with hospitalized patients with HF were close to the median, which may suggest that there are no prevalence differences in terms of recruitment settings. 

Second, depressive symptoms were measured using four different self-report questionnaires in the 10 studies. The measures used in the studies have been validated and widely used to assess depressive symptoms among cardiac patients [24], and the validity and reliability of the Korean version of each measure have also been established [14,16,18,19]. Because each measure was used in only one to three studies, we were unable to explore whether the reported depression rates varied by the measure used to assess depression. Although previous systematic reviews showed no significant differences among various self-report measures in detecting depression in primary care settings [25] or in patients with HF in Western countries [6], future research is needed to examine whether similar results are found with non-Western patients. We also found inconsistencies in applying cut-off points across the studies reviewed. Depending on the population studied, using a liberal cut-off point (e.g., indicating mild depression) may improve sensitivity and specificity in identifying depressed individuals more than using a conservative cut-off point (e.g., indicating moderate depression) or vice versa [6]. Therefore, it may be beneficial to report prevalence rates using both cut-offs unless the optimal cut-off point is known for the selected measure. Moreover, the two studies that used the CES-D [19,22] reported different numbers of items in the instrument and used different scoring methods. Therefore, the prevalence rates reported in the studies need to be interpreted with caution. We also recommend that the established scoring method for each measure be used consistently in future studies.

Given the high prevalence of clinical depression among patients with HF in Korea and worldwide, patients need to be screened for depressive symptoms. Because of its brevity and well-established psychometric properties in cardiac patients, the American Heart Association Prevention Committee [26] has recommended the PHQ-9 as a screening tool for depression in cardiac patients. Its Korean version has also demonstrated good reliability and validity in the Korean population [27]. As recommended by the American Heart Association Prevention Committee, the two-step approach (i.e., having the patient fill out the PHQ-2 inquiring about depressed mood and anhedonia and then applying all nine items of the PHQ-9 when the answer to either or both questions is “yes”) may be especially useful in clinical settings. Routine and ongoing assessment of depressive symptoms at HF clinics is recommended to identify newly developed depressive symptoms and monitor changes in existing symptoms, and therefore to improve patients’ clinical outcomes [10,23].

Three studies [13,16,21] reported the percentage of patients with HF who were on antidepressants, which ranged between 14% and 26%. These numbers were far below the depression prevalence rates reported in each of the studies, which ranged from 31% to 48%. Other treatment modalities are also known to be effective for depression among cardiac patients, including cognitive behavioral therapy, exercise programs, and interpersonal psychotherapy [24,26]. These treatment modalities were also recommended in the evidence-based treatment guidelines for depression developed by the Clinical Research Center for Depression in Korea [28]. However, none of the reviewed studies reported the percentage of depressed patients with HF utilizing non-pharmacological treatments. In addition, none of the non-pharmacological interventions have been tested in depressed patients with HF in Korea. Patients with depression typically prefer non-pharmacological treatment, and this is particularly true among Koreans [28]. Therefore, future research is needed to establish the effectiveness of these non-pharmacological interventions among depressed patients with HF in Korea, and evidence-based non-pharmacological treatments should be offered as a treatment option for those who are depressed. 

Depression is a complex disorder with multidimensional symptoms (i.e., cognitive-affective symptoms, such as sadness, anhedonia, hopelessness, irritability, difficulty with concentration, guilt, and suicidal ideation; and somatic symptoms, such as fatigue, sleep disturbances, psychomotor agitation or retardation, and appetite changes) [29]. While previous studies have shown that somatic depressive symptoms are more frequently reported in non-Western cultures than in Western cultures [11], the current literature on depression and HF in Korea does not provide sufficient information to examine cultural differences in the presentation of depressive symptoms. To examine whether similar cultural differences exist in the presentation of depressive symptoms among patients with HF, it is recommended that future studies report not only total scores, but also subscale scores of the depression measures.

A few limitations need to be addressed regarding this integrative review. Following the review guidelines [12], we excluded grey literature (i.e., unpublished theses and dissertations, abstracts, and conference proceedings). However, this may have introduced publication bias. In addition, despite our efforts to ensure a thorough literature search using six electronic databases with search terms in both English and Korean, it is possible that some articles containing findings relevant to this review may have been missed because the terms used in our search did not match with the keywords in their titles or abstracts.

## 5. Conclusions 

To date, only a limited number of studies exist regarding depressive symptoms among patients with HF in Korea. Due to the heterogeneity in study samples and measures of depression, as well as methodological problems, the findings need to be interpreted with caution. However, our review highlights a number of areas that need to be addressed in future research and clinical practice with HF patients in Korea. More research studies are needed on depressive symptoms among patients with HF in Korea, especially longitudinal studies. It is also important to use a validated measure with an established cut-off score to assess depressive symptoms. In addition, future studies need to assess how depression in patients with HF is managed in Korea, including pharmacological and non-pharmacological treatments. These data will provide a foundation for additional studies to test the effectiveness of interventions for reducing depressive symptoms and improving clinical outcomes of patients with HF in Korea. Clinicians working with patients with HF need to pay careful attention to identifying depressive symptoms and to providing proper treatment for depressed patients.

## Figures and Tables

**Table 1:** Summary of studies reporting depressive symptoms among patients with heart failure in Korea.

Study	Sample Size (*N*)	Data Collection Period	Patient Characteristics			Patient Recruitment Setting	Assessment of Depressive Symptoms			
			Age (Mean ± SD)	Female (%)	NYHA Class III or IV (%)		Measure	Score (Mean ± SD)	Cut-Off Score Used	Clinically Depressed (%)
Chu et al., 2012 [17]	133	October 2010–February 2011	61.4 ± 13.6	26.3	10.0	Out-patient	PHQ-9	4.1 ± 4.4	≥5	36.8
Lee, H. et al., 2015 [19]	169	April 2012–September 2013	62.9 ± 8.7	19.5	9.5	Out-patient	CES-D	15.8 ± 10.0	≥16	43.2
Lee, S. et al., 2005 [22]	105	May 2004–January 2005	Not reported (≥65 years 73.3%)	51.4	11.4 (No class IV)	Out-patient	CES-D	Mean = 30.18 SD = not reported	Not used	Not reported
Son et al., 2012 [18]	134	February 2010–May 2010	67.8 ± 8.8	63.4	26.1 (No class IV)	Out-patient	GDS-SF	6.9 ± 3.8	≥5	67.9
Song, 2009 [13]	254	September 2005–December 2006	62 ± 14	42.9	52.4	Out-patient	BDI	17.9 ± 9.6	≥16	48.4
Song et al., 2006 [20]	260	September 2005–December 2005	62.2 ± 13.1	41.9	66.2	In-patient	BDI	18.3 ± 10.1	Not used	Not reported
Song et al., 2009 [21]	231	Not reported	63 ± 13	42.4	Not reported	In-patient	BDI	Not reported	≥17	44.6
Song et al., 2014 [14]	243	Not reported	61 ± 14	39.1	46.9	In-patient	BDI	14.7 ± 9.5	≥17	36.6
Song et al., 2015 [15]	297	September 2011–December 2012	64.4 ± 9.8	28.3	51.2	Out-patient	PHQ-9	6.0 ± 5.5	≥10	23.6
Song et al., 2015 [16]	232	March 2012–March 2013	66 ± 8	33.6	45.2	Out-patient	PHQ-9	6.9 ± 5.7	≥10	31.0

BDI = Beck Depression Inventory; CES-D = Center for Epidemiologic Studies Depression Scale; GDS-SF = Geriatric Depression Scale-Short Form; NYHA = New York Heart Association; PHQ-9 = Patient Health Questionnaire 9 items.

## Abbreviations

The following abbreviations are used in this manuscript:

HF	Heart failure
CINAHL	Cumulative Index to Nursing and Allied Health Literature
KMbase	Korean Medical Database
RISS	Research Information Sharing Service
KISS	Korean Studies Information Service System
NYHA	New York Heart Association
BDI	Beck Depression Inventory
CES-D	Center for Epidemiologic Studies Depression Scale
GDS-SF	Geriatric Depression Scale-Short Form
PHQ-9	Patient Health Questionnaire
